# Lymphnode tuberculosis in a 4-year-old boy with relapsed ganglioneuroblastoma: a case report

**DOI:** 10.1186/s12879-018-3016-x

**Published:** 2018-03-05

**Authors:** Karoline van de Loo, Stefan Balzer, Colin R. MacKenzie, Thomas M. Boemers, Monika Ortmann, Jörg Schaper, Arndt Borkhardt, Hans-Jürgen Laws, Michaela Kuhlen

**Affiliations:** 1 Department of Paediatric Oncology, Medical Faculty, Haematology and Clinical Immunology, Centre for Child and Adolescent Health, University of Duesseldorf, Moorenstr. 5, 40225 Duesseldorf, Germany; 2 Institute of Medical Microbiology and Hospital Hygiene, University Hospital of Duesseldorf, Heinrich-Heine University, Moorenstr. 5, 40225 Duesseldorf, Germany; 3 Department of Pediatric Surgery and Pediatric Urology, Children’s Hospital, Amsterdamer Str. 59, 50735 Cologne, Germany; 4 Institute of Pathology, University Hospital of Cologne, Kerpener Str. 62, 50937 Cologne, Germany; 5 Department of Diagnostic and Interventional Radiology, Medical Faculty, University of Duesseldorf, Moorenstr. 5, 40225 Duesseldorf, Germany

**Keywords:** *Mycobacterium tuberculosis* infection, Lymphnode tuberculosis, Relapsed ganglioneuroblastoma, Interferon gamma release assay

## Abstract

**Background:**

*Mycobacterium tuberculosis (M. tuberculosis)* disease is a generally well-known problem among immunocompromised adults and children. In pediatric oncology, only few cases of *M. tuberculosis* disease are reported so far.

**Case presentation:**

We report a case of concomitant lymphnode tuberculosis in a 4-year-old German boy with relapsed ganglioneuroblastoma. 18 months after the initial diagnosis, relapse with new paravertebral lesions and new lesions in the left lower lobe of the lung and in the perihilar lymphnodes suspicious of metastases of the ganglioneuroblastoma were detected. While relapse in the tumor was confirmed, unexpectedly, pathologic examination revealed morphological diagnosis of lymphnode tuberculosis. The boy was of German background without previous history of tuberculosis exposure. Both, antituberculostatic and relapse treatment were immediately initiated. Three months on, MRI revealed regressive findings in the lung and lymphnodes and partial response in the tumor. The patient underwent second MiBG therapy and haploidentical stem cell transplantation.

**Conclusion:**

The diagnosis of lymphnode tuberculosis in a 4-year-old German boy with relapsed ganglioneuroblastoma was only made by chance, but most likely saved his life. Pediatric oncologist should be aware of tuberculosis as the incidence might increase over time and the timely diagnosis of a potentially preventable *M. tuberculosis* disease is irreplaceable. Further studies are needed to explore the incidence of *M. tuberculosis* infections and the value of IGRA, testing for latent tuberculosis infection prior to chemotherapy in children with underlying malignancies.

## Background

Childhood tuberculosis (TB) is a well-known disease among immunocompromised children in high-burden countries [1]. In Europe, the awareness of TB is very low, although during the period 2000 to 2009, nearly 40.000 cases of *Mycobacterium tuberculosis* (*M. tuberculosis*) disease in children were reported by the countries of the European Union [2].

Infectious complications in pediatric oncology patients are quite common. However, *M. tuberculosis* disease in these children is rarely considered, most likely due to the putative low-incidence. Particularly, in children who live in low-burden countries such as Germany and who present without a history of exposure, the differential diagnosis of *M. tuberculosis* disease is often neglected [2]. In such low-incidence countries one third of the pediatric cases are of foreign origin, of whom many cases still are without a history of exposure [2]. Screening children for latent TB infection (LTBI) is not part of the standard initial diagnostic work up in most German pediatric oncology departments. Up to date, there are only few reports on *M. tuberculosis* disease concerning children with oncologic or hematologic diseases [3].

Even in the setting of autologous hematopoietic stem cell transplantation (HSCT) in children suffering from neuroblastoma, infectious complications most commonly are of minor importance. In a cohort of 73 children suffering from neuroblastoma who underwent autologous HSCT, there were 12 cases of bacteremia or fungemia, but no death related to these infectious complications [4]. Thus, the awareness of infectious complications in neuroblastoma patients is quite low. Additionally, so far, to the best of our knowledge, there is no report of *M. tuberculosis* disease in a patient suffering from neuroblastoma.

We describe the case of a 4-year-old boy with relapsed high-risk ganglioneuroblastoma in whom the resection of suspicious lymphnodes revealed the diagnosis of lymphnode tuberculosis instead of neuroblastoma metastases 18 months after the initial diagnosis.

## Case presentation

**Fig. 1:**
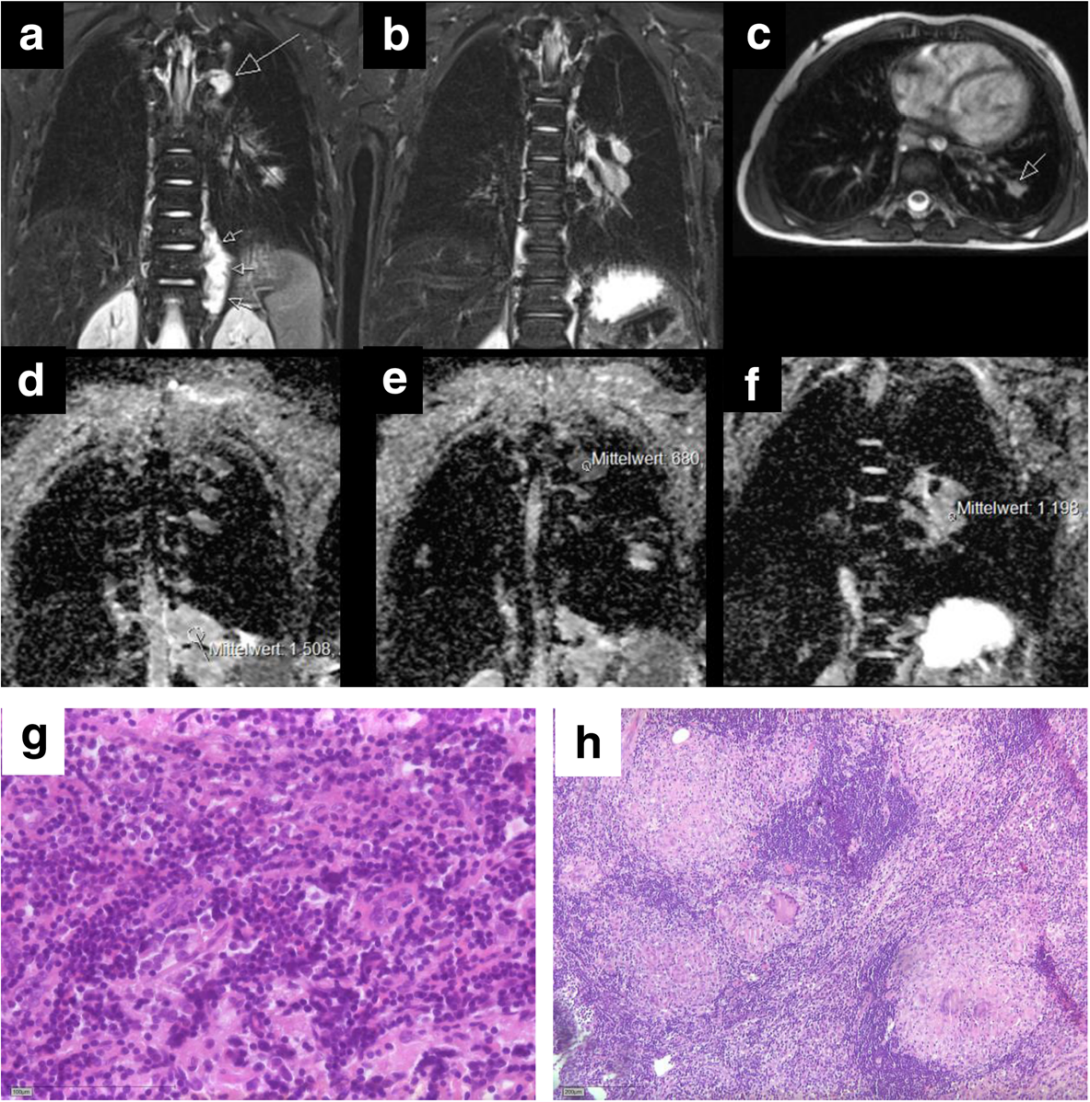
**a**-**c** STIR cor and Trufisp axial. **a** Known residual tumor left paravertebral TH10–12 (short arrows) and new tumor manifestation at the level of TH5 left paravertebral (long arrow). **b** Left hilar lymphadenopathy. **c** Pulmonary lesion in left lower lobe (short arrow). **d**-**f** ADC-maps of the different mediastinal lesions. **d** known primary tumor. **e** recurrent tumor. **f** left hilar lymphadenopathy. **g**-**h** Hematoxylin and eosin stains. **g** High power field of the neuroblastoma-relapse, Schwannian stroma-poor, undifferentiated according to the INPC classification, immunohistochemically positive for nb84a, synaptophysin and CD56, not shown) (HE, bar 100 μm). **h** Granulomatous lymphadenitis with epithelioid appearance and multinucleated giant cellls (Langhans-type) suspicious of flourishing lymphnode tuberculosis (even without proof of mycobacteria-specific DNA) (HE, bar 200 μm.). Noncaseating granuloma

A 4-year-old German boy with stage 4 ganglioneuroblastoma was treated with chemotherapy, metaiodobenzylguanidine (MIBG) therapy, autologous HSCT and irradiation according to the German Pediatric Oncology Group Neuroblastoma High risk 2004 protocol. 18 months after initial diagnosis, relapse with new paravertebral lesions was detected by MRI and MIBG scintigraphy. In addition, new lesions in the left lower lobe of the lung and in the perihilar lymphnodes suspicious of metastases of the ganglioneuroblastoma were detected by MRI but did not present in MIBG scintigraphy (Fig. 1a-f). Biopsy of the paravertebral tumor was performed and the suspicious perihilar lymphnodes were resected. While relapse in the tumor was confirmed, surprisingly, pathologic examination revealed granulomatous lymphadenitis with epithelioid appearance and Langhans-type multinucleated giant cells suspicious of flourishing lymphnode tuberculosis instead of metastases of the ganglioneuroblastoma. The captured granulomatous lymphadenitis was noncaseating (Fig. G-H). In addition, an interferon-gamma release assay (IGRA) yielded positive, whereas polymerase chain reaction (PCR) and cultures could not detect any mycobacteria. However, diagnosis of peripheral lymphnode tuberculosis was made based on histomorphology and IGRA. Notably, no history of tuberculosis exposure could be identified. The boy did not suffer from fever, weight loss or night sweats. He was not vaccinated with BCG.

The patient underwent 2 months of triple therapy with pyrazinamide, rifampin and isoniazid and 3 months of rifampin and isoniazid treatment. For neuroblastoma relapse, chemotherapeutical treatment according to the RIST-relapsed Neuroblastoma-2011 protocol was administered.

Three months after initiating the antituberculostatic treatment and relapse chemotherapy, MRI showed a partial remission of the paravertebral lesions and a nearly complete remission of the lesions in the lung and lymphnodes.

Meanwhile, the patient underwent a second MiBG therapy and haploidentical stem cell transplantation. Actually, he is undergoing anti-gd2 antibody therapy.

## Discussion and conclusions

Here we present a case of lymphnode tuberculosis in a boy with relapsed ganglioneuroblastoma. The diagnosis could have easily been missed. The *M. tuberculosis* infection occurred simultaneously with the relapse of the ganglioneuroblastoma and was therefore initially misdiagnosed as neuroblastoma lymphnode metastases. Only by chance, these lymphnodes were resected. To subsequently confirm suspicious of pulmonary tuberculosis, resection and additional histopathological and microbiological examination would have been necessary. Indeed we refrained from doing so as treatment would not have changed. Instead, as any delay in treatment for relapse of the ganglioneuroblastoma as well as of tuberculosis could have caused life-threatening complications, we immediately initiated combination therapy.

Under both, the antituberculostatic and chemotherapeutical treatment, all lesions decreased in size with the perihilar lymphnodes and pulmonary changes showing the best response. Thus, it is most likely that also the lesion in the left lower lobe of the lung was pulmonary tuberculosis.

In our case, the infection with *M. tuberculosis* could not be confirmed microbiologically. Reported culture confirmation rates vary widely. In the European Union, only 16.9% of all pediatric tuberculosis cases reported from 2000 to 2009 were confirmed by cultures [2]. Nevertheless, the combination of the histopathological findings suggestive for tuberculosis and the positive IGRA are most likely for *M. tuberculosis* infection. No caseating granuloma was detected, which is most likely due to an initial stage of inflammation of TB infection. The IGRA has the highest sensitivity and specificity to test for LTBI. Noteworthy, in children under 5 years, the IGRA shows a sensitivity of 83% only [5]. However, the same analysis showed a specifitciy of nearly 96% for the positive IGRA in children with active *M. tuberculosis* disease [5]. Moreover, PCR results are not inconsistent with *M. tuberculosis* infection as this was performed on a formalin-fixed, paraffin-embedded samples, the latter compromising the sensitivity of the PCR. Summing up, in our case, the diagnosis of lymphnode tuberculosis is most likely, however, not proven. To definitely proof lymphnode tuberculosis, confirmation of *M. tuberculosis* by culture or at least the detection of *M. tuberculosis* by PCR would have been needed.

To the best of our knowledge, this is the first report on *M. tuberculosis* disease in a child suffering from neuroblastoma. It demonstrates that establishing the diagnosis of *M. tuberculosis* disease particularly in immunocompromised children remains challenging. Due to the unspecific symptoms and high percentage of lacked microbiological confirmation, the real incidence of this disease is unknown [2]. There are only a few studies reporting single cases or small case series on tuberculosis in pediatric hematology and oncology. In these, the diagnosis is often delayed because of the uncharacteristic symptoms with the most prominent symptom being prolonged fever of unknown origin [3, 6–11]. Due to the uncommonness, the uncharacteristic symptoms as well as the challenges in confirming the diagnosis, there is a high possibility that *M. tuberculosis* disease is missed and, thus, is responsible for unexplained infectious deaths in children with cancer. Almost all studies reporting on *M. tuberculosis* disease in pediatric hematology and oncology relate to children suffering from leukemia or those who underwent HSCT [6–11]. There is only one report on children with other malignancies suffering from tuberculosis [3]. There might be a higher awareness of infectious complications in these patients compared to children with solid malignancies. In addition, leukemia itself and the use of high-dosed steroids might attribute to the higher rate of infectious complications with *M. tuberculosis* in these patients [8].

In our case, the origin of the tuberculosis infection remains somehow indeterminable as the boy was of German background without previous history of tuberculosis exposure on the one side and reconstituted immune system on the other side. However, the coincidental diagnosis of *M. tuberculosis* infection prior to starting chemotherapy and proceeding to HSCT very likely saved his life.

We encountered a case of concomitant lymphnode tuberculosis in a 4-year-old German boy with relapsed ganglioneuroblastoma. Early diagnosis and immediate initiation of treatment most likely saved his life.

As the incidence of tuberculosis in pediatric oncology might not only be underestimated, but also increase over time due to immigration of families from low-income countries, pediatric oncologist should be aware of this infection as a timely diagnosis of a potentially preventable TB disease is irreplaceable.

Further studies are needed to explore the incidence of TB infections and the value of IGRA testing for LTBI prior to chemotherapy in children with underlying malignancies.

## Abbreviations

HSCT: Hematopoietic stem cell transplantation;
IGRA: Interferon-gamma release assay;
LTBI: Latent TB infection;
*M. tuberculosis*: *Mycobacterium tuberculosis*;
MIBG: Metaiodobenzylguanidine;
PCR: Polymerase chain reaction;
TB: Tuberculosis
